# Are all LGBTQI+ patients white and male? Good practices and curriculum gaps in sexual and gender minority health issues in a Dutch medical curriculum

**DOI:** 10.3205/zma001315

**Published:** 2020-03-16

**Authors:** Maaike Muntinga, Juliëtte Beuken, Luk Gijs, Petra Verdonk

**Affiliations:** 1 Amsterdam UMC-VUmc, School of Medical Sciences, Amsterdam Public Health research institute, Department of Medical Humanities, Amsterdam, The Netherlands; 2 Amsterdam UMC-VUmc, Center of Expertise on Gender Dysphoria, Amsterdam, The Netherlands

**Keywords:** LGBTQI+, curriculum screening, marginalized populations, sexual and gender diversity, medical education

## Abstract

**Objectives: **People marginalized based on their sexual and gender identity face specific health risks and experience barriers to culturally competent care. Insight into how Dutch medical schools address LGBTQI+ health-related learning objectives is scarce. We therefore examined how LGBTQI+ health issues are integrated in the Amsterdam UMC-VUmc medical curriculum by evaluating the year-two course ‘Sex, Sexuality and Relationships’ for LGBTQI+ content.

**Methods/Design: **We examined written course content (course syllabus, lecture notes, and course literature) of the 2016-2017 course. We used a framework for essential LGBTQI+ content in medical education and an intersectional approach to examine which LGBTQI+ themes and subthemes were addressed.

**Results: **Several essential LGBTQI+ health issues were adequately addressed and integrated into the Amsterdam UMC-VUmc curriculum, but we also identified curriculum gaps. The needs of patients with lesbian, bisexual, or gender non-conforming identities were marginally addressed, and issues related to intersections of minoritized sexual and gender identities with other aspects of diversity such as ethnicity, age and class remained unexplored. The course discussed gender and sexuality as fixed and mainly binary constructs, and only addressed biomedical explanatory models of sex, gender and sexuality.

**Discussion and conclusion: **The absence of complex patient identities in relation to sex, gender and sexuality does not adequately prepare students to provide LGBTQI+ responsive care. If not designed and taught competently, LGBTQI+-related curriculum content may reproduce bias and stereotypes, and contribute to a medical climate where both LGBTQI+ patients, students, and doctors conceal their identities. Further implementation of LGBTQI+ health issues is required in (continuing) medical education to secure culturally competent clinical environments. Educational research is needed to understand how medical education contributes to marginalization of LGBTQI+ identities and thus, to health disparities.

## Zusammenfassung

**Zielsetzung: **Menschen, die aufgrund ihrer sexuellen und geschlechtsspezifischen Identität marginalisiert werden, sind spezifischen Gesundheitsrisiken ausgesetzt und erleben Barrieren für eine kulturell kompetente Pflege. Ein Einblick in die Art und Weise, wie niederländische medizinische Hochschulen LGBTQI+-bezogene Gesundheits-Lernziele behandeln ist rar. Wir haben daher untersucht, wie LGBTQI+-Gesundheitsthemen in den medizinischen Lehrplan der Amsterdam UMC-VUmc integriert werden, indem wir den zweijährigen Kurs „Sex, Sexualität und Beziehungen“ für LGBTQI+-Inhalte evaluierten.

**Methoden und Design: **Wir haben die schriftlichen Kursinhalte (Lehrplan, Vorlesungsfolien und Kursliteratur) der Lehrveranstaltung 2016-2017 begutachtet. Wir verwendeten wesentliche LGBTQI+-Inhalte in der medizinischen Ausbildung als Rahmen und einen intersektionalen Ansatz, um zu untersuchen, welche LGBTQI+-Themen und Unterthemen angesprochen wurden.

**Ergebnisse: **Einige grundlegende LGBTQI+-Gesundheitsthemen wurden angemessen behandelt und in den Amsterdamer UMC-VUmc-Lehrplan integriert. Allerdings haben wir auch Lücken im Lehrplan identifiziert. Die Bedürfnisse von Patient*innen mit lesbischen, bisexuellen oder geschlechtervarianten Identitäten wurden nur marginal berücksichtigt. Probleme, die durch die Überlappung von minorisierten sexuellen und geschlechtsspezifischen Identitäten mit anderen Aspekten der Diversität wie Ethnizität, Alter und Klasse entstehen, blieben unerschlossen. Der Kurs behandelte Geschlecht und Sexualität als feste und vor allem binäre Konstrukte und befasste sich lediglich mit biomedizinischen Erklärungsmodellen von Geschlecht, Gender und Sexualität.

**Diskussion und Schussfolgerung: **Das Fehlen komplexer Identitäten von Patient*innen in Bezug auf Geschlecht, Gender und Sexualität, bereitet die Studierenden nicht ausreichend darauf vor, eine LGBTQI+-gerechte Behandlung anzubieten. Wenn LGBTQI+-bezogene Lehrplaninhalte nicht kompetent konzipiert und gelehrt werden, können sie Bias und Stereotypen reproduzieren und zu einem medizinischen Klima beitragen, in dem sowohl LGBTQI+-Patient*innen, Studierende als auch Ärzt*innen ihre Identität verbergen. Die weitere Implementierung von LGBTQI+-Gesundheitsthemen ist in der (weiteren) medizinischen Ausbildung erforderlich, um ein kulturkompetentes klinisches Umfeld zu sichern. Bildungsforschung ist notwendig, um zu verstehen, wie die medizinische Ausbildung zur Marginalisierung der LGBTQI+-Identitäten und damit zu gesundheitlichen Ungleichheiten beiträgt.

## 1. Introduction

### 1.1. Problem 

People minoritized based on their sexual or gender identity face particular health risks and barriers in access to care, and therefore have specific health and care needs [1], [2], [3]. Health needs of LGBTQI+ (lesbian, gay, bisexual, transgender, queer, intersex and other identities such as gender non-binary) people have been identified in areas as end of life care [4], mental health [5], reproductive and sexual health and family planning [6], smoking cessation [7], [8], oral care [9], primary care and public health and prevention [10]. In addition, LGBTQI+ health concerns and experiences in the health care systems are informed not only by sexual and gender identity, but also by intersections with other aspects of identity, such as age, race and migrant status [10], [11], [12]. A growing body of research shows that quality of care for LGBTQI+ people is compromised, and that LGBTQI+ people experience health disparities [1], [13], [14], [15], [16], [17]. For instance, patients face barriers entering the health care system or gaining access to tailored, culturally competent care [13], [17], [18], [19], [20], [21]. Providers are generally unaware of the impact of sexual and gender identities on every day (health) experiences [22], and some refuse to address gender and sexuality issues because of their own value systems or negative attitudes towards LGBTQI+ patients [23], [24]. Although existing research into the experiences of LGBTQ+-patients focuses primarily on white, middle class individuals [12], evidence suggest LGBTQI+ patients from underrepresented groups or underserved communities are particularly vulnerable to health inequities based on additional disparities related to multiple marginalizations [25]. It is essential that physicians have the knowledge and skills to provide tailored care to LGBTQI+ patients of all backgrounds.

Health education is key to the advancement of health equity, and future health professionals should be trained in providing culturally competent and sensitive services (e.g. [2], [16], [26], [27], [28]. Unfortunately, medical students are insufficiently prepared to tailor care to LGBTQI+ communities [15], [29], [30]. Unmet learning needs of health professions students have been reported regarding, for instance, LGBTQI+ responsive sexual and reproductive care [6], transgender and intersex health [31], [32], and health care for older LGBTQI+ patients [14], [26], [31], [33]. Therefore, recent years have seen the publication of several development frameworks and implementation resources for medical educators [3], [34]. In 2015, the Association of American Medical Colleges (AAMC) published an eight-domain competency plan, and comprehensive implementation packages for medical educators and administrators, including group assignments and lecture slides [34]. Despite these efforts, curricular integration of sexual and gender diversity is often poor: the amount of curriculum time assigned to LGBTQI+ issues is limited[3], [35], [36], and studies report LGBTQI+ content is met with resistance by educators [35]. The latter points at the institutional climate as one barrier to successfully implement sexual and gender diversity issues in undergraduate and graduate medical education [14], [26], [34], [35], [37], [38]. 

So far, little to no insight exists into the extent to which Dutch medical schools address sexual and gender diversity learning objectives. Scarce evidence suggests that implementation is limited and that material is not structurally embedded in educational programs [39], [40]. For instance, a previous screening of the formal, undergraduate curriculum at Amsterdam UMC-VUmc School of Medical Sciences (VUmc SMS) for diversity-related content identified a lack of material addressing LGBTQI+ health across intersections of ethnicity, age, class and ability [40]. In addition, few Dutch studies have explored intersections of LGBTQI+ experiences and health beyond HIV/AIDS and transitional care. In 2001, the Netherlands was the first country in the world to legalize same sex marriage, and has been lauded internationally for its tolerance toward diverse sexual identities [41]. However, despite increased visibility and growing societal acceptance of non-normative sexual and gender identities, LGBTQI+ people in the Netherlands are still confronted with negative reactions from their environment (such as bullying), experience verbal, physical and sexual harassment and violence, and are more often victim of a crime [42]. Considering these statistics, the lack of scientific attention for LGBTQI+ health in a country famous for its progressive politics seems paradoxical.

#### 1.2. Aim of the study

Given the observed gaps in knowledge about medical schools teach about LGBTQI+ health, the aim of our study was to gain-an in-depth insight in the way in which LGBTQ+ health issues are currently addressed in the formal undergraduate curriculum of Amsterdam UMC-VUmc School of Medical Sciences (VUmc SMS), in particular in an undergraduate course about sexual and reproductive health, “Sex, Sexuality and Relationships”. We based our choice to screen this course on the expectation that a course on sexuality and relationships would be most likely to feature LGBTQI+ learning objectives. Using a framework for essential LGBTQI+ topics in medical education as a reference [3], we discuss the integration of LGBTQI+ health issues in the formal VUmc SMS curriculum, and identify good practices as well as curriculum gaps. 

## 2. Methods

### 2.1. Study design: Intersectionality-based course evaluation

Using an intersectionality-based approach, we evaluated the VUmc SMS course “Sex, Sexuality and Relationships” for LGBTQI+ content. Intersectionality theory aims to analyze and understand human difference, and calls attention to underrepresented and marginalized social groups (e.g. [43], [44]). An intersectional approach to health research, policy or practice takes into account how social identities are mutually constitutive in shaping health experiences and outcomes, as well as how power operates across institutions to drive health inequalities and inequities [45], [46], [47], [48], [49]. 

In health research and medical discourse, the category “LGBTQI+” is often used as an uncomplicated and homogenous group. However, in relation to LGBTQI+ health, sociocultural identity markers such as gender, ethnicity, age, religious background or spirituality, or whether one has a mental or physical disability all shape LGBTQI+ experiences, and thus account for diversity among patients. To provide good care to all LGBTQI+ patients, medical students should be trained to adequately take this diversity into account. When carrying out the course screening, we therefore not only investigated which LGBTQI+ content was addressed in the course, but also whether course material addressed intersections of LGBTQI+ identity with other aspects of identity in relation to health. We acknowledge that by our use of the acronym LGBTQI+ when referring to people with non-hegemonic bodies, sexualities or genders, we potentially reproduce essentialist understandings of minoritized communities. Nevertheless, because our intention is to draw attention to the specific needs of these communities and to how these needs should be addressed by health professions curricula, we use the acronym strategically, an approach referred to as “strategic essentialism” [50]. 

#### 2.2. Setting: Diversity at VUmc SMS

VUmc SMS is a medical school connected to a large teaching hospital and one of eight medical schools in the Netherlands. The medical school traditionally has a diverse student body, with approximately one in three students having a non-Western migrant background. VUmc SMS values diversity, equity and inclusion, which is expressed in its organizational policies and practices [40], [51]. For instance, the school-funded subcommittee of the local medical student association, D.O.C.S (Diversity, Openness, Culture, Students), is dedicated to create an inclusive campus climate and empower students with non-Western or migration backgrounds. However, so far, diversity efforts at VUmc SMS have mainly been directed at aspects of ethnic diversity. LGBTQI+ medical students are not officially organized within the institution, and their experiences have not been studied. 

In addition to the organizational focus on diversity, diversity issues have been increasingly receiving attention within the VUmc SMS curriculum. For instance, in recent years, less visible or invisible identities have been brought into medical students’ awareness by means of a vertically integrated learning pathway “Interculturalization and Diversity”, which is part of an overarching ‘professional behavior’ track within the VUmc SMS curriculum [40], [52]. Aim of this pathway is to mainstream diversity issues throughout the curriculum by building students’ competencies related to interaction with patients with diverse backgrounds and identities. In addition to knowledge and skills, learning objectives include reflexivity – for instance, regarding how students’ own social identities inform their value systems, and how this could impact the doctor-patient relationship or create barriers in providing high quality and effective care for all patients (e.g. [51], [53]). The learning pathway consists of lectures, practicals and required reading (a Dutch-language textbook “Cultural diversity in healthcare” [54]. An intersectionality approach guides the pathways’ learning objectives, shapes curriculum content and the design of curriculum evaluations, outcomes of which are used to further improve and tailor curricular content [40]. Cultural competencies are tested twice during the undergraduate phase by means of OSCEs. 

#### 2.3. The course ‘Sex, Sexuality and Relationships’

So far, the “Interculturalisation and Diversity” pathway has not yet implemented learning objectives that explicitly center on LGBTQI+ issues. Instead, learning objectives related to sexual and gender identity are addressed separately and horizontally in the course “Sex, Sexuality and Relationships”, a four-week course focusing on sexuality, fertility and reproduction, that takes place in the second year of the undergraduate curriculum. At the time of the study, two authors (MM and PV) were involved in the implementation, coordination and delivery of “Interculturalisation and Diversity” content. One author (LG) was involved as a coordinator and instructor for the “Sex, Sexuality and Relationships” course. 

“Sex, Sexuality and Relationships” is divided into four themes. Week one centers on “sex development, the development of feeling male or female and the development of (sexual) relationships”; week two centers on “female F, atypical/abnormal sexual attractions and orientations”; week three centers on “aspects of both masculine and feminine sexuality”; and week four centers on mainly “male sexuality”. Specific topics addressed within these themes are sex differentiation, development of gender identity, atypical sex differentiation (i.e. intersex conditions, also referred to in medical contexts as Disorders/Differences of Sexual Development, DSD), gender dysphoria, (the psychophysiological study of) sexual arousal, anatomy and embryology of the genitalia, female and male sexuality (with specific emphasis on sexual dysfunction and genital problems); atypical sexual attractions, puberty, the menstrual cycle, menopause and aging in males, Sexually Transmitted Infections (STI’s), professional boundaries in physician-patient interaction, discussing sexuality in a professional context and taking a sexual history. The course’s LGBTQI+ related learning objectives are presented in table 1 (Tab. 1).

#### 2.4. Data collection and analyses 

We screened all written content of the 2016-2017 course “Sex, Sexuality and Relationships” for LGBTQI+ themes. Documents were retrieved from a digital learning environment and the university medical library, and included the course syllabus (sixteen student assignments), nineteen lecture notes, and course literature (scientific articles and textbooks). Access to the course’s digital learning environment was obtained with permission from the course coordinator (LG) by JB, who was a research intern at the time. Access to the library was obtained by MM through their position as faculty staff. 

To analyze what content was addressed and how, we used a framework for essential LGBTQI+ content in undergraduate medical education proposed by Obedin-Maliver and colleagues [3]. In their 2011 JAMA article, the authors distinguish sixteen essential LGBTQI+ related topics for the required medical curriculum: sexual orientation, HIV, gender identity, sexually transmitted infections (STI’s), safer sex, DSD/intersex, barriers to care, mental health issues, LGBT adolescents, coming out, unhealthy relationships/intimate partner violence (IPV), substance use, chronic disease risk, sex-reassignment surgery (SRS), body image, and transitioning [3]. 

The analysis was carried out by JB and MM and consisted of two parts. First, we used the Obedin-Maliver framework as a template to investigate

1. which topics were addressed, and
2. to which extent they were addressed.

We considered a topic “explicitly addressed” when it was featured as a learning objective in the course syllabus, when it was addressed in a lecture, or when students were asked to apply knowledge about the topic (for instance derived from required course reading) to a patient case in a study assignment. We considered a topic “marginally addressed” when it was only addressed in the course reading. We considered a topic “not addressed” when it was neither addressed in a lecture or study assignment, nor in the course literature. Second, we explored whether material in which LGBTQI+ identities were explicitly addressed explored intersections of LGBTQI+ identity with other aspects of diversity – such as ethnicity, age, class or ability – in relation to a particular topic or health issue. Based on the outcomes of this analysis, we identified good practices and opportunities for improvement. 

## 3. Results

First, we present which topics were addressed in the course, and how they were addressed. Next, we report curriculum gaps. 

### 3.1. LGBTQI+ topics in the course 

Of the sixteen topics included in Obedin-Maliver’s framework, only one essential aspect of LGBTQI+ health (body image) remained completely unaddressed in lectures, study assignments and literature. All other aspects were addressed in study material; however, the degree to which and how they were addressed varied. For instance, some topics (such as needs and experiences of LGBTQI+ adolescents, including coming out, and IPV) were briefly mentioned in the course reading, but were not elaborated on in lecture slides or study assignments. Attachment 1 presents an overview of which topics were addressed in the course, how they were addressed (in the literature, in a lecture or in a study assignment), and which topics were marginally addressed or absent. 

The LGBTQI+-related themes featured in the course were addressed both from a biomedical, psychosocial and ethical perspective. LGBTQI+ health outcomes were addressed in a study assignment and two scientific articles (i.e. [55], [56]), which focused on health needs of men who have sex with men (MSM) in primary care (addressing aspects such as HIV/STD’s and substance use) and on mental health (discussing minority stress, stigma and discrimination) [55], [56]. A study assignment addressed mental and physical health issues of sexual minority men by presenting a case study of a male, gay-identified patient (study assignment “Homosexuality”). In addition to biomedical explanatory models for homosexual orientation, another good practice was the incorporation of study material that encouraged students to critically consider the relationship between hormones, sex chromosomes and sexual orientation, reflect on the validity of a purely biomedical explanation for homosexuality, explore moral perspectives on studying the etiology of homosexuality, and reflect on the American Psychiatric Association definition of “homosexuality”. Gender diversity and intersex were addressed in a lecture on medical gender reassignment surgery, and in a study assignment on sex differentiation, including etiologies and DSD prevalence. Students were asked to reflect on whether and how to reveal the diagnosis DSD to the parents and the child with DSD, and on the appropriateness of genital surgery in in newborns with ambiguous genitalia. Course literature included information about psychological determinants and processes that influence gender development, and the difference between sex, gender identity and gender presentation.

#### 3.2. Curriculum gaps

Several essential learning objectives remained unaddressed or under-addressed. Unaddressed were topics related to body image and identity, and formal course material did not explicitly contain learning objectives related to LGBTQI+ inclusive practices, for instance regarding (sexual) history taking and counseling. Although learning objectives of a practical about sexual history taking included a focus on students exploring their own norms and values around sexuality, whether and how this included norms and values about sexual and gender diversity was unclear, as was the extent to which specific needs of LGBTQI+ patients related to sensitivity in clinical interactions and communication were addressed. Explanatory models of sexual and gender diversity and LGBTQI+ health inequities were primarily addressed from a biomedical perspective. For instance, students were asked to reflect on biomedical models of homosexuality, but no alternative models and paradigms were offered, and students were not asked to reflect on and critically consider broader LGBTQI+ disparities and their (sociocultural and historical) origins in relation to exclusion and stigmatization.

We found that physical, mental and social health issues were addressed predominantly from the perspective of gay MSM, and that intersections of LGBTQI+ identity with other aspects of diversity such as ethnicity, age or class remained unexplored. For example, health and care needs of patients with lesbian identities or women who have sex with women (WSW), as well as of bisexual/pansexual and gender non-conforming patients, were either marginally addressed or not addressed. Trans health and care needs were not addressed beyond endocrinological and surgical transition, and barriers to care for LGBTQI+ patients were addressed in relation to MSM, but not specifically for other marginalized sexual and gender identities, including intersex/DSD patients. Moreover, the course did not address objectives and material related to the ways in which other aspects of identity such as ethnicity, age and class shape health experiences and needs of LGBTQI+ patients. For example, the homosexual patient with mental health issues featured in the study assignment about “homosexuality” is a white, highly-educated male of Dutch national origin. Finally, gender was addressed as a fixed and (mostly) binary construct, and students were not invited to consider health issues of people with non-binary or fluid identities, or question the biological and social categories “male” and “female”. 

## 4. Discussion

We used Obedin-Maliver and colleagues’ 2011 framework and an intersectional approach to evaluate which LGBTQI+-related content was addressed in the Amsterdam UMC-VUmc undergraduate course Sex, Sexuality and Relationships. Although the course did address a range of LGBTQI+ issues, we identified curriculum gaps. Several LGBTQI+-related issues and their intersections with for instance ethnicity, age or class were unaddressed or marginally addressed, there was limited visibility of health and care needs of identities on the LGBTQI+ spectrum other than the cisgender gay male, and gender and sexuality were discussed as fixed and binary concepts. As a result, the perspectives, needs and vulnerabilities of people with complex or multiple non-hegemonic identities were absent from the course material.

### 4.1. LGBTQI+ knowledge gaps in medical education 

While attention for sexual and gender diversity in society is growing and the range of sexual and gender identities is expanding, medical schools are slow to incorporate new knowledge into their curricula [35], [57]. In line with our findings, previous studies have shown that time dedicated to LGBTQI+ themes is limited, that several learning objectives are structurally overlooked, and that health needs of people with non-normative sexual and gender identities remain underexplored [58]. Gaps related to LGBTQI+ issues in medical curricula are problematic for several reasons. First, students miss out on acquiring essential thematic knowledge and skills necessary to optimally tailor care to patients of all orientations and backgrounds. Lack of student preparedness could lead to LGBTQI+ patients experiencing barriers to accessing care or not receiving the care they need [31], [59]. It is imperative that students know how to use inclusive language and adopt sensitive communication practices, because care for LGBTQI+ patients can be compromised when they do not feel safe enough in care environments to disclose their orientation to their health professionals [18], [59], [60], [61]. 

Second, a comprehensive understanding of patient needs and preferences in the age of personalized medicine requires learning beyond etiology, epidemiology, disease prevalence and treatment models, but should also involve insight in contextual factors that influence patient outcomes. Aspects of sociocultural identity such as ethnicity, age and religious beliefs influence LGBTQI+ cultures and experiences, including their experiences in the health care system [62]. Cultural competence, then, should involve in-depth understanding of complexity and how it relates to diversity in care needs, clinical presentations and treatment preferences. Not accounting for such complexity in curricular material potentially sustains clinical bias based on assumed homogeneity of social groups, reproduces stereotypes, and overlooks the impact of sociocultural (risk) environments on lived experiences in relation to health, illness and disease [63], [64]. In addition, categories of sexual and gender identity as well as biological sex are increasingly understood as spectral, fluid and unstable [65], [66], [67] which advocates for the use of sociocultural explanatory models alongside biomedical ones [68], [69], [70], [71], [72].

Finally, our findings show that an LGBTQI+ friendly cultural climate and the adoption of tolerance toward sexual and gender minorities as a collective Dutch value do not necessarily translate into curricular innovation. It is quite possible that the ideology of equality in Dutch medical discourse translates into the assumption that a patient’s sexual and gender identity is irrelevant medical information, unless clinically relevant – for instance, when it is a social determinant of disease (for instance, in high-income countries, in the case of HIV/AIDS). In the traditionally positivist biomedical climate, “clinically relevant” often refers to biomedical information and cues, and not to social ones [69], [73]. The notion that knowing about a patients’ sexual and gender identity is fundamentally irrelevant for diagnosis and clinical decision making has been previously described, for instance by Roberson (2014) in his work on experiences of medical students with LGBTQI+ curricular content. Robertson refers to this idea as the “irrelevance narrative” [74]. The irrelevance narrative might become internalized by medical students, including students who identify as LGBTQI+ themselves, and cause marginalized sexual and gender identities to become invisible in educational and clinical environments [64], [74], [75]. Such invisibility obscures the specific health risks and needs of LGBTQI+ patients, which sustains health inequities. In addition, it contributes to a climate where LGBTQI+ students might choose to conceal their identities because of fear of discrimination, lack of support, and cultural norms including norms around medical professionalism [35], [76].

#### 4.2. Strengths and limitations 

This is the first study to evaluate the extent to which LGBTQI+ health topics are integrated in a Dutch medical curriculum. Because VUmc SMS has explicitly committed itself to curricularization of diversity topics and its efforts have been recognized as leading in the country, our findings might point at a larger need across medical schools in the Netherlands to realize and optimize integration of LGBTQI+ health content.

Although the course Sex, Sexuality and Relationships is likely the course to feature most learning objectives related to sexual and gender diversity, we did not screen other courses for LGBTQI+ content, which might have resulted in us having overlooked topics. Moreover, we only examined the written, formal curriculum, and have no insight in how LGBTQI+ topics are taught in the classroom. Because individual teacher engagement and competency is essential to adequately address LGBTQI+ health issues without reproducing stereotypes or perpetuate bias, classroom observational research can contribute to a better understanding of the informal and hidden curriculum, so as to gain insight in not only what is taught, but also what is learned.

## 5. Conclusion

Although the formal Amsterdam UMC-VUmc curriculum covered a wide range of LGBTQI+-related issues, our screening of the undergraduate course Sex, Sexuality and Relationships revealed opportunities for improvement, in particular related to identities beyond the white gay male and the incorporation of changing scientific and societal understandings of categories of sex, gender and sexuality as spectral, fluid and diverse. Intersectional approaches to curriculum design and evaluation can help gain better insight in which identities and topics are overlooked in course material and identify potential sources of clinical bias. To understand how medical education might contribute to marginalization of LGBTQI+ identities and thus to health disparities, a closer examination of the informal and hidden curriculum is essential. Further implementation of sexual and gender diversity topics is required in (continuing) medical education to safeguard clinical environments responsive to the needs of LGBTQI+ patients of all backgrounds. 

## Authors contributions

All authors have substantially contributed to the article and approved the form and content of the manuscript.

## Competing interests

The authors declare that they have no competing interests. 

## Supplementary Material

Outcomes of the course screening, organized according to Obedin-Maliver’s framework for essential LGBTQI+-related topics in medical education.

## Figures and Tables

**Table 1:**
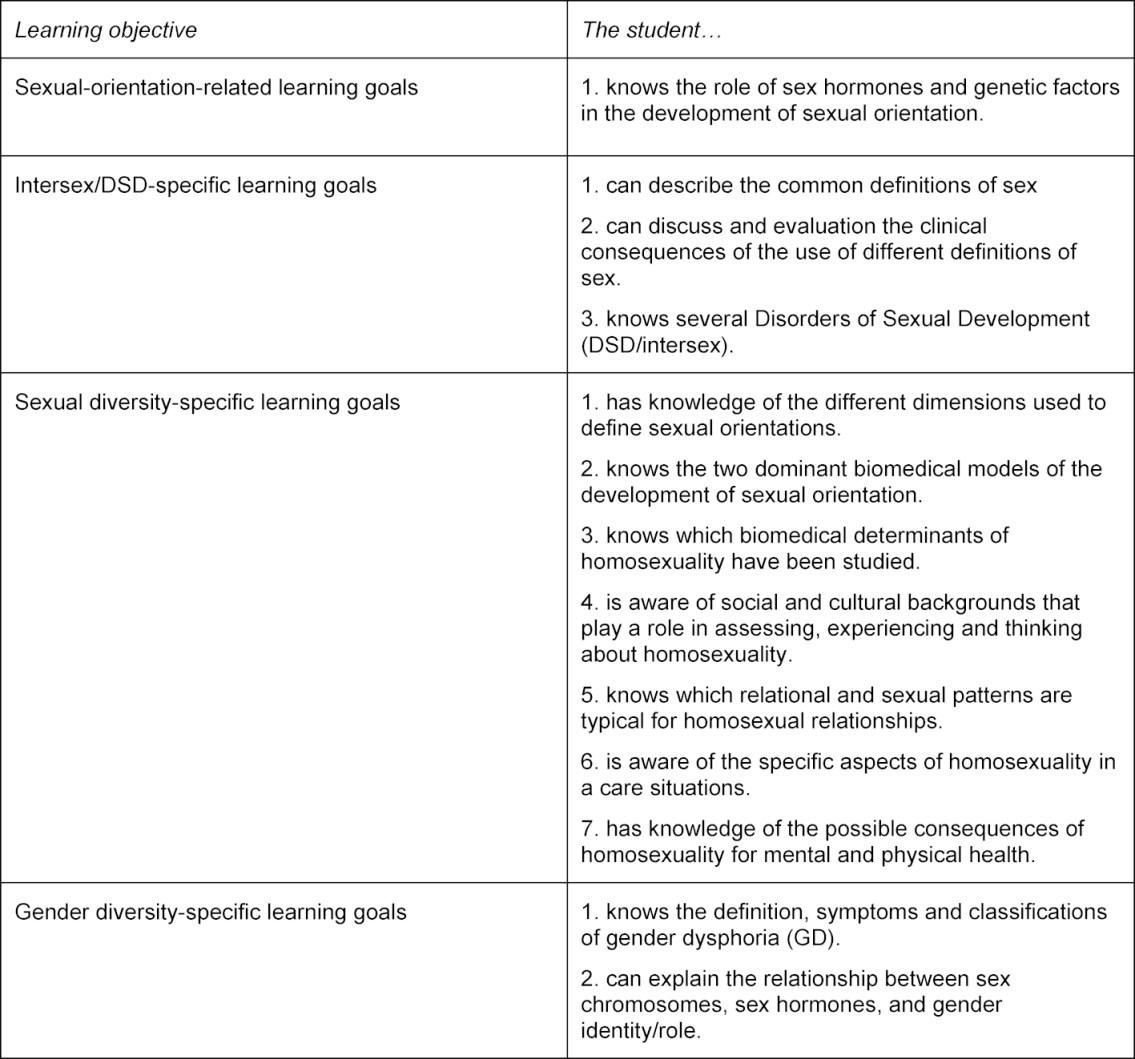
Learning objectives of the VUmc SMS undergraduate course ‘Sex, Sexuality and Relationships’ (source: course syllabus)

## References

[1] Daniel H, Butkus R, Health and Public Policy Committee of American College of Physicians (2015). Lesbian, gay, bisexual, and transgender health disparities: executive summary of a policy position paper from the American College of Physicians. Ann Intern Med.

[2] Fredriksen-Goldsen KI, Hoy-Ellis CP, Goldsen J, Emlet CA, Hooyman NR (2014). Creating a vision for the future: Key competencies and strategies for culturally competent practice with lesbian, gay, bisexual, and transgender (LGBT) older adults in the health and human services. J Gerontol Soc Work.

[3] Obedin-Maliver J, Goldsmith ES, Stewart L, White W, Tran E, Brenman S, Wells M, Fetterman DM, Garcia G, Lunn MR (2011). Lesbian, gay, bisexual, and transgender-related content in undergraduate medical education. JAMA.

[4] Griebling TL (2016). Sexuality and aging: a focus on lesbian, gay, bisexual, and transgender (LGBT) needs in palliative and end of life care. Curr Opin Support Palliat Care.

[5] Rodgers SM (2017). Transitional Age Lesbian, Gay, Bisexual, Transgender, and Questioning Youth: Issues of Diversity, Integrated Identities, and Mental Health. Child Adolesc Psychiatr Clin N Am.

[6] Walker K, Arbour M, Waryold J (2016). Educational Strategies to Help Students Provide Respectful Sexual and Reproductive Health Care for Lesbian, Gay, Bisexual, and Transgender Persons. J Midwifery Womens Health.

[7] Berger I, Mooney-Somers J (2017). Smoking Cessation Programs for Lesbian, Gay, Bisexual, Transgender, and Intersex People: A Content-Based Systematic Review. Nicotine Tob Res.

[8] Bruce Baskerville N, Wong K, Shuh A, Abramowicz A, Dash D, Esmail A, Kennedy R (2018). A qualitative study of tobacco interventions for LGBTQ+ youth and young adults: overarching themes and key learnings. BMC Public Health.

[9] Russell S, More F (2016). Addressing Health Disparities via Coordination of Care and Interprofessional Education: Lesbian, Gay, Bisexual, and Transgender Health and Oral Health Care. Dent Clin North Am.

[10] Bostwick WB, Meyer I, Aranda F, Russell S, Hughes T, Birkett M, Mustanski Bl (2014). Mental health and suicidality among racially/ethnically diverse sexual minority youths. Am J Public Health.

[11] Balsam KF, Molina Y, Beadnell B, Simoni J, Walters K (2011). Measuring multiple minority stress: the LGBT People of Color Microaggressions Scale. Cultur Divers Ethnic Minor Psychol.

[12] LaVaccare S, Diamant AL, Friedman J, Singh KT, Baker JA, Rodriguez TA, Cohen SR, Dary FY, Pregler J (2018). Healthcare Experiences of Underrepresented Lesbian and Bisexual Women: A Focus Group Qualitative Study. Health Equity.

[13] Bonvicini KA (2017). LGBT healthcare disparities: What progress have we made?. Patient Educ Couns.

[14] Giffort DM, Underman K (2016). The relationship between medical education and trans health disparities: a call to research. Soc Compass.

[15] Keuroghlian AS, Ard KL, Makadon HJ (2017). Advancing health equity for lesbian, gay, bisexual and transgender (LGBT) people through sexual health education and LGBT-affirming health care environments. Sexual Health.

[16] Lim FA, Brown D, Kim SJ (2014). CE: Addressing health care disparities in the lesbian, gay, bisexual, and transgender population: a review of best practices. Am J Nurs.

[17] Rubin R (2015). Minimizing health disparities among LGBT patients. JAMA.

[18] Colpitts E, Gahagan J (2016). "I feel like I am surviving the health care system": understanding LGBTQ health in Nova Scotia, Canada. BMC Public Health.

[19] Roberts TK, Fantz CR (2014). Barriers to quality health care for the transgender population. Clin Biochem.

[20] Rosario VA (2015). Cultural competence and LGBT issues in psychiatry. Psychiatr Times.

[21] Valentine SE, Shipherd JC (2018). A systematic review of social stress and mental health among transgender and gender non-conforming people in the United States. Clin Psychol Rev.

[22] Daley A, MacDonnell JA (2015). 'That would have been beneficial': LGBTQ education for home-care service providers. Health Soc Care Community.

[23] Dorsen C (2012). An integrative review of nurse attitudes towards lesbian, gay, bisexual, and transgender patients. Can J Nurs Res.

[24] Lim FA, Hsu R (2016). Nursing Students' Attitudes Toward Lesbian, Gay, Bisexual, and Transgender Persons: An Integrative Review. Nurs Educ Perspect.

[25] Institute of Medicine, Board on the Health of Select Populations, Gay, Bisexual, and Transgender Health Issues and Research Gaps and Opportunities Committee of Lesbian (2011). The health of lesbian, gay, bisexual, and transgender people: Building a foundation for better understanding.

[26] Cannon SM, Shukla V, Vanderbilt AA (2017). Addressing the healthcare needs of older Lesbian, Gay, Bisexual, and Transgender patients in medical school curricula: a call to action. Med Educ Online.

[27] Khalili J, Leung LB, Diamant AL (2015). Finding the perfect doctor: identifying lesbian, gay, bisexual, and transgender-competent physicians. Am J Public Health.

[28] Sawning S, Steinbock S, Croley R, Combs R, Shaw A, Ganzel T (2017). A first step in addressing medical education Curriculum gaps in lesbian-, gay-, bisexual-, and transgender-related content: The University of Louisville Lesbian, Gay, Bisexual, and Transgender Health Certificate Program. Educ Health (Abingdon).

[29] White W, Brenman S, Paradis E, Goldsmith ES, Lunn MR, Obedin-Maliver J, Stewart L, Tran E, Wells M, Chamberlain LJ, Fettermann DM, Garcia G (2015). Lesbian, gay, bisexual, and transgender patient care: Medical students' preparedness and comfort. Teach Learn Med.

[30] Zelin NS, Hastings C, Beaulieu-Jones BR, Scott C, Rodriguez-Villa A, Duarte C, Calahan C, Adami AJ (2018). Sexual and gender minority health in medical curricula in new England: a pilot study of medical student comfort, competence and perception of curricula. Med Educ Oonline.

[31] Korpaisarn S, Safer JD (2018). Gaps in transgender medical education among healthcare providers: A major barrier to care for transgender persons. Rev Endocr Metab Disord.

[32] Liang JJ, Gardner IH, Walker JA, Safer JD (2017). Observed deficiencies in medical student knowledge of transgender and intersex health. Endocr Pract.

[33] Sekoni AO, Gale NK, Manga-Atangana B, Bhadhuri A, Jolly K (2017). The effects of educational curricula and training on LGBT-specific health issues for healthcare students and professionals: a mixed-method systematic review. J Int AIDS Soc.

[34] Hollenbach AD, Eckstrand KL, Dreger AD (2014). Implementing curricular and institutional climate changes to improve health care for individuals who are LGBT, gender nonconforming, or born with DSD: a resource for medical educators.

[35] Davy Z, Amsler S, Duncombe K (2015). Facilitating LGBT medical, health and social care content in higher education teaching. Qual Res Educ.

[36] Müller A (2013). Teaching lesbian, gay, bisexual and transgender health in a South African health sciences faculty: addressing the gap. BMC Med Educ.

[37] Eckstrand KL, Lunn MR, Yehia BR, Hadland SE, Yehia BR, Makadon HJ (2017). Applying Organizational Change to Promote Lesbian, Gay, Bisexual, and Transgender Inclusion and Reduce Health Disparities. LGBT Health.

[38] Snowdon S (2013). Recommendations for enhancing the climate for LGBT students and employees in health professional schools.

[39] Dautzenberg M (2015). Anders dan normaal. LGBTQ-patienten in de Nederlandse zorg.

[40] Muntinga M, Krajenbrink V, Peerdeman S, Croiset G, Verdonk P (2016). Toward diversity-responsive medical education: taking an intersectionality-based approach to a curriculum evaluation. Adv Health Sci Educ Theory Pract.

[41] Ayoub P, Paternotte D (2014). LGBT activism and the making of Europe: a rainbow Europe?.

[42] Boss EM, Felten H (2017). Handreiking LHBT-emancipatie: feiten en cijfers op een rij.

[43] Cho S, Crenshaw KW, McCall L (2013). Toward a field of intersectionality studies: Theory, applications, and praxis. J Women Cult Society.

[44] Crenshaw K, Bergen RK, Edleson JL, Renzetti CM (2005). Mapping the Margins: Intersectionality, Identity Politics, and Violence against Women of Color (1994). Violence against woman: Classic papers.

[45] Dhamoon RK, Hankivsky O, Hankivxky O (2011). Why the theory and practice of intersectionality matter to health research and policy. Health inequities in Canada: Intersectional frameworks and practices.

[46] Hancock AM (2007). Intersectionality as a normative and empirical paradigm. Politic Gender.

[47] Hankivsky O (2014). Intersectionality 101.

[48] Hankivsky O, Reid C, Cormier R, Varcoe C, Clark N, Benoit C, Brotman S (2010). Exploring the promises of intersectionality for advancing women's health research. Int J Equity Health.

[49] Springer KW, Hankivsky O, Bates LM (2012). Gender and health: relational, intersectional, and biosocial approaches. Soc Sci Med.

[50] Epstein S (2008). Inclusion: The politics of difference in medical research.

[51] Bruin N, Muntinga M, Verdonk P (2018). Preaching to the choir? Sociodemographic differences in medical students' evaluation of an undergraduate diversity training module. MedEdPublish.

[52] Mak-van der Vossen M, Peerdeman S, Kleinveld J, Kusurkar R (2013). How we designed and implemented teaching, training, and assessment of professional behaviour at VUmc School of Medical Sciences Amsterdam. Med Teach.

[53] Verdonk P, Abma T (2013). Intersectionality and reflexivity in medical education research. Med Educ.

[54] Wolffers IN, van der Kwaak A, van Beelen N (2013). Culturele diversiteit in de gezondheidszorg.

[55] Herek GM, Garnets LD (2007). Sexual orientation and mental health. Annu Rev Clin Psychol.

[56] Makadon HJ, Mayer KH, Garofalo R (2006). Optimizing primary care for men who have sex with men. JAMA.

[57] McCann E, Brown M (2018). The inclusion of LGBT+ health issues within undergraduate healthcare education and professional training programmes: A systematic review. Nurse Educ Today.

[58] Donald CA, DasGupta S, Metzl JM, Eckstrand KL (2017). Queer frontiers in medicine: A structural competency approach. Acad Med.

[59] Baldwin A, Dodge B, Schick V, Herbenick D, Sanders SA, Dhoot R, Fortenberry JD (2017). Health and identity-related interactions between lesbian, bisexual, queer and pansexual women and their healthcare providers. Cult Health Sex.

[60] Brooks H, Llewellyn CD, Nadarzynski T, Pelloso FC, Guilherme FDS, Pollard A, Jones CJ (2018). Sexual orientation disclosure in health care: a systematic review. Br J Gen Pract.

[61] Durso LE, Meyer IH (2013). Patterns and predictors of disclosure of sexual orientation to healthcare providers among lesbians, gay men, and bisexuals. Sex Res Social Policy.

[62] Eliason MJ, Chinn PL (2017). LGBTQ cultures: What health care professionals need to know about sexual and gender diversity.

[63] Heyes CJ, Thachuk A (2015). Queering know-how: clinical skill acquisition as ethical practice. J Bioeth Inq.

[64] Baker K, Beagan B (2014). Making assumptions, making space: An anthropological critique of cultural competency and its relevance to queer patients. Med Anthropol Q.

[65] Lykens JE, LeBlanc AJ, Bockting WO (2018). Healthcare Experiences Among Young Adults Who Identify as Genderqueer or Nonbinary. LGBT Health.

[66] Ainsworth C (2015). Sex redefined. Nature.

[67] Joel D, Tarrasch R, Berman Z, Mukamel M, Ziv E (2014). Queering gender: studying gender identity in 'normative'individuals. Psychol Sex.

[68] Kuper A, D'eon M (2011). Rethinking the basis of medical knowledge. Med Educ.

[69] Kuper A, Veinot P, Leavitt J, Levitt S, Li A, Goguen J, Schreiber M, Richardson L, Whitehead CR (2017). Epistemology, culture, justice and power: non-bioscientific knowledge for medical training. Med Educ.

[70] Hyde JS, Bigler RS, Joel D, Tate CC, van Anders SM (2019). The future of sex and gender in psychology: Five challenges to the gender binary. Am Psychol.

[71] Hammack PL, Frost DM, Hughes SD (2019). Queer intimacies: A new paradigm for the study of relationship diversity. J Sex Res.

[72] Fausto-Sterling A (2019). Gender/Sex, Sexual Orientation, and Identity Are in the Body: How Did They Get There?. J Sex Res.

[73] Kuper A, Messinger A, Rangel C, Cartmill C, Martimianakis T, Whitehead C (2016). What is Considered "legitimate" Medical Education Research? A Discourse Analysis of the Journal Medical Education. Med Educ.

[74] Robertson WJ (2017). The Irrelevance Narrative: Queer (In) Visibility in Medical Education and Practice. Med Anthropol Q.

[75] Müller A (2018). Beyond 'invisibility': queer intelligibility and symbolic annihilation in healthcare. Cult Health Sex.

[76] Mansh M, White W, Gee-Tong L, Lunn MR, Obedin-Maliver J, Stewart L, Goldsmith E, Brenman S, Tran E, Wells M, Fettermann D, Garcia G (2015). Sexual and gender minority identity disclosure during undergraduate medical education:"In the closet" in medical school. Acad Med.

