# Supplementary material for: Are all LGBTQI+ patients white and male? Good practices and curriculum gaps in sexual and gender minority health issues in a Dutch medical curriculum
Source: GMS J Med Educ. 2020 Mar 16;37(2):Doc22. doi: 10.3205/zma001315 (PMC7171358; doi:10.3205/zma001315)
Supplement: Outcomes of the course screening, organized according to Obedin-Maliver’s framework for essential LGBTQI+-related topics in medical education. [file JME-37-22-s-001.pdf]

Attachment 1: *Outcomes of the course screening, organized according to Obedin-Maliver's framework for essential LGBTQI+-related topics in medical education.*

LGBTQI+-related topic	Health issue or topic	Addressed	Marginally addressed	Not addressed
Sexual orientation		• Biomedical determinants and explanatory models for homosexual orientation (sex chromosomes, hormones) *, **<br>• Own moral perspectives on research into etiology of homosexuality ***<br>• Prevalence homosexuality *		• Difference between sexual orientation, identity and behavior<br>• Perspectives on conceptualization of sexual orientation (e.g. essentialist or binary conceptualization vs constructivist or dynamic/fluid conceptualization)<br>• Challenges in researching sexual orientation<br>• LGBTQI+-competency, responsivity and sensitivity, e.g. language use and communication strategies
Gender identity		• Determinants and processes that influence the development of gender identity from psychological perspective *, **<br>• Gendered aspects of sexual functioning *, **	• Psychopathology **<br>• Difference between sex, gender identity and gender presentation/ gender role behavior **	• Perspectives on conceptualization and understandings of gender (e.g. essentialist or binary understanding vs constructivist or dynamic/fluid understanding)
DSD/intersex		• Determinants of sex-differentiation (genetic, neuro-		• Perspectives on conceptualization of sex (e.g. essentialist or binary

		endocrinologal perspective) *, **, ***<br>• Prevalence and incidence of DSD in the Netherlands *<br>• DSD types and clinical profiles, treatment options and trajectories *<br>• Ethical aspects of disclosure to patient and family ***<br>• Ethical and biomedical aspects of clinical decision making **, ***		conceptualization vs constructivist or dynamic/complex conceptualization)
LGBTQI+-specific health issues		In general:<br>• Students are asked to mention mental and physical health issues in homosexual men and women in study assignment ‘homosexuality’***	In general:<br>• Minority stress model, psychopathological model **	In general:<br>• Fertility and pregnancy<br>• Risk environments in relation to sexual and gender minorities
	HIV		• HIV-risk and risk factors in MSM **	• HIV-risk, risk factors, risk environments in WSW, and trans or non-binary people
	STI (other than HIV)		• STI risk and risk factors in MSM **	• STI-risk, risk factors and risk environments in WSW, and trans or non-binary people
	Safer Sex		• In article Makadon et al. (2006) **	• Sexual practices and cultures other than ‘MSM’ not

Attachment 1 to: *Muntinga M, Beuken J, Gijs L, Verdonk P. Are all LGBTQI+-patients white and male? Good practices and curriculum gaps in sexual and gender minority health issues in a Dutch medical curriculum. GMS J Med Educ. 2020;37(2):Doc22. DOI: 10.3205/zma001315, URN: urn:nbn:de:0183-zma0013151 Online available from: https://www.egms.de/en/journals/zma/2020-37/zma001315.shtml*

Attachment 1: *Outcomes of the course screening, organized according to Obedin-Maliver's framework for essential LGBTQI+-related topics in medical education.*

				addressed, incl. heterogeneity of cultures and practices among MSM
	Mental health issues		• In article Herek et al. (2007)**	• Not addressed for BTQI+-dentities
	Substance use		• In article Makadon et al. (2006) **	• Not addressed for LBTQI+-identities
	Chronic disease risk		• Briefly in relation to screening for hepatitis A, B or STI-related cancer in MSM in article Makadon et al. (2006) **	• Not addressed for LBTQI+-identities
	Unhealthy relationships/IPV		• Briefly in relation to screening IPV in men who have sex with men in article Makadon et al. (2006) **	• Prevalence and particularities of IPV in LGBTQI+-relationships not addressed
T	Body image			• Not addressed
	Coming out		• Briefly in article Makadon et al. (2006) **<br>• In article Herek et al. (2007)**	• Communicationor counseling strategies for guiding coming out trajectories in practice not addressed
Trans health-specific issues	Sex reassignment surgery	• Surgical aspects of medical transition, surgical complications, contra-indications *		• Patient perspectives and variation in whishes (i.e. partial wishes) not addressed

Attachment 1: *Outcomes of the course screening, organized according to Obedin-Maliver's framework for essential LGBTQI+-related topics in medical education.*

	Transition	• Medical aspects of transition addressed (treatment trajectory, surgical interventions and complications) *		• Aspects of social transition pre and post- surgery not addressed<br>• Trans-specific mental and physical health issues not addressed<br>• Gender-affirmative and trans-sensitive communication and practice not addressed
LGBTQI+ adolescents			• Briefly in article Makadon et al. (2006) about coming out **	• Strategies for practice not addressed<br>• LBTQI+-adolescents not addressed
Barriers to care			• In article Makadon et al. (2006), for MSM**	• Not specifically addressed for LBTQI+-patients

DSD=disorders of sexual development; HIV=human immunodeficiency virus; IPV = intimate partner violence ; LGBTQI+=lesbian, gay, bisexual, transgender, queer, intersex, and other non-normative sexual and gender identities; MSM = men who have sex with men; WSW = women who have sex with women; STI = sexually transmitted infections;
*addressed in lecture
** addressed in literature
*** addressed in group study assignment
